# Biopreservation of Refrigerated Mackerel (*Auxis thazard*) Slices by Rice Starch-Based Coating Containing Polyphenol Extract from *Glochidion wallichianum* Leaf

**DOI:** 10.3390/foods11213441

**Published:** 2022-10-30

**Authors:** Paramee Chumsri, Worawan Panpipat, Lingzhi Cheong, Atikorn Panya, Natthaporn Phonsatta, Manat Chaijan

**Affiliations:** 1 Food Technology and Innovation Research Center of Excellence, School of Agricultural Technology and Food Industry, Walailak University, Nakhon Si Thammarat 80160, Thailand; 2 Zhejiang-Malaysia Joint Research Laboratory for Agricultural Product Processing and Nutrition, College of Food and Pharmaceutical Science, Ningbo University, Ningbo 315211, China; 3 Food Biotechnology Research Team, Functional Ingredients and Food Innovation Research Group, National Center for Genetic Engineering and Biotechnology (BIOTEC), Pathumthani 12120, Thailand

**Keywords:** fish, shelf-life, edible coating, refrigeration, plant extract

## Abstract

Both microbial decomposition and oxidative deterioration contribute to the qualitative degradation of fresh or minimally preserved fish, which negatively impacts the shelf-life of fish, especially those with dark flesh like mackerel. It is becoming more typical to use edible coatings to preserve the freshness of fish products. Herein, the effects of a rice starch (RS) based coating incorporated with dried crude, aqueous Mon-pu (*Glochidion wallichianum*) leaf extract (MPE) at varying concentrations (0, 0.02, 0.1, 0.5, and 1.0% *w*/*w*) on the quality characteristics of mackerel (*Auxis thazard*) slices during storage at 4 °C were investigated. Uncoated slices had a shelf-life of 6 days, whereas samples coated with RS and 0.5% MPE extended the shelf-life to 9 days by keeping the overall microbiological quality below the permitted level of 6 log CFU/g. The changes in thiobarbituric acid reactive substances (TBARS; <2 mg malondialdehyde equivalent/kg), propanal content, heme iron degradation, myoglobin redox instability, and surface discoloration (a* value and total color difference; ΔE) can all be delayed by this coating condition. Additionally, the RS-MPE coating can maintain the sensory quality of refrigerated mackerel slices and preserve the textural property (water holding capacity and hardness), as well as postpone the development of an off-odor as indicated by lowered contents of total volatile base-nitrogen (TVB-N; not exceeding the acceptable limit of 25 mg/100 g) and trimethylamine (TMA; not exceeding the acceptable limit of 10 mg/100 g). Therefore, a biopreservative coating made of RS and MPE, especially at 0.5%, can be employed to extend the shelf-life of refrigerated mackerel slices up to 9 days.

## 1. Introduction

Raw fish slices like sushi and sashimi are popular worldwide ready-to-eat prepared foods [1]. Sushi and sashimi are often made with fresh dark-fleshed fish (such as tuna and mackerel), and the color of the fish affects how well it is received [2]. Furthermore, both microbial and chemical actions can cause the deterioration in the quality of fish flesh [3,4,5]. Microbial decomposition [6,7,8] and oxidative deterioration [9,10,11,12], particularly in pelagic dark-fleshed fish, are the main drivers of fish quality loss in fresh or minimally preserved fish. In addition, dark-fleshed fish are more susceptible to discoloration due to the redox instability of myoglobin when kept at a cold temperature [10,13,14]. In Southern Thailand, the frigate mackerel (*Auxis thazard*) is a significant dark-fleshed fish species with a large yield that can be utilized to make sashimi or raw fish slices. Unsaturated fatty acids and myoglobin are abundant in its muscle. Thus, during storage, Frigate mackerel’s lipid oxidation and discoloration are easily possible, which substantially impedes its processing and use [15]. According to Lahreche et al. [16], frigate mackerel fillets have a rather low shelf-life when stored in refrigerators, necessitating the development of ways to increase it. Fish is typically kept refrigerated to retain its quality [17]. However, some disadvantages of cold storage include dehydration, textural hardness, nutritional loss, and decreased protein extractability. Finding new ways to lower quality loss and prolong the shelf-life of fish fillets is of importance given the rising demand for refrigerated fresh fish fillets. Several processing processes may be used to increase the raw fish flesh’s shelf-life [5]. Notably, quality control procedures significantly reduce the capacity of spoilage bacteria to proliferate and reduce the rate of deterioration [18,19]. In order to enhance the quality of fresh tuna slice and lengthen its shelf-life, a number of techniques have been used, including ultra-low temperature storage [13], the application of natural antioxidants [2], and coating with an edible film containing plant extract [20]. Additionally, stepwise chilling of 0.5 °C every 18 h was used to maintain mackerel suitable for sushi or sashimi in a supercooled state [21]. The need to create bio-safe techniques for fish fillet preservation is growing [22]. 

It has become increasingly popular to utilize edible coatings to preserve products from adverse environmental effects and increase their shelf-life [23,24,25]. An edible coating, also known as a film-forming solution or dispersion, is an edible substance that is applied thinly and directly on food. It is typically administered by spraying or immersing the item with the solution [26,27]. The film serves as a barrier against gases and water vapor, which reduces oxygen levels and helps to regulate microbial, enzyme, and oxidative reactions of the coated product during storage [25]. The composition of edible coatings affects their effectiveness and stability. Since they are naturally occurring polymers, polysaccharides such as cellulose, starch, pectin, alginates, chitosan, and others are frequently utilized for this purpose [28,29]. Starch is a suitable polysaccharide for food coating due to its filmogenic capability, ease of accessibility, and low cost [30]. Starch can be acquired from a variety of sources, including cereals (wheat, corn, or rice), tubers (cassava or potato), and legumes (pea) [31]. Fruits, vegetables, and other items are routinely coated with starch to increase shelf-life [25] but starch coating is still uncommon on fish slices. According to a recent study by Martins et al. [32], oregano oil added to a rice starch (RS) film increased the shelf-life of marine catfish (*Genidens genidens*) and prevented the proliferation of bacteria such *Escherichia coli*, *Salmonella enteritidis*, and coagulase-positive staphylococci.

The advantage of adding various additives to the coating is that it enables a delayed release of these substances from the film. These additives are typically proteins, chitosan, bacteriocins, essential oils, and plant extracts. Each of these substances affects lipid oxidation, proteolytic degradation, and microbiological decay in a distinct way [33]. In order to satisfy consumer demands, natural antioxidant and antibacterial components have been substituted for synthetic additions in the preservation of chilled fish fillets [4]. Natural preservatives are growing increasingly popular among consumers as they become more knowledgeable about food safety [34]. Antioxidants can minimize oxidation by decreasing or delaying the production of free radicals, whereas antimicrobial compounds can suppress bacteria, boosting food safety [34]. The fish industry has also studied plant extracts as preservatives [35]. Particularly, leaf extracts of polyphenols have been well studied [7]. The various polyphenol components have biological activities including antioxidant and antibacterial capabilities [36]. According to Pan et al. [37], fish fillets treated with tea polyphenols had a longer shelf-life and showed less bacterial population and total volatile base-nitrogen (TVB-N) than control fillets. *Glochidion wallichianum* Mull. Arg., also known as Mon-Pu in Thai, is an edible native plant found in Thailand that possesses considerable antioxidant components in its young leaves [38]. These leaves can be eaten fresh and prepared similarly to other local vegetables. The Mon-Pu leaf extracts (MPE) have potent antioxidant effects and a high phenolic content [39,40,41]. The antioxidant activity of MPE was also demonstrated by Tangkanakul et al. [42] and Panpipat et al. [43] using DPPH^●^/ABTS^●+^ inhibition and ferric reducing antioxidant power (FRAP). The polyphenolic compounds and other phytochemicals (e.g., β-carotene, lutein, terpenoids, ascorbic acid, and tocopherol) are the active antioxidant components of MPE [39,40]. The most prevalent antioxidant in MPE is polyphenolic, namely gallic acid, which is related to its considerable antioxidant potential [44]. Overall, MPE’s antioxidant characteristics in vitro [43], in an o/w emulsion [42], and in a sausage model system [38] make it a practical, consumer-friendly technique for reducing oxidative instability in fish slices. The RS can be applied directly to the surface of the mackerel slice to serve as an edible coating that protects the product, enhances its quality, and lengthens its shelf-life. As a consequence, the goal of this study was to ascertain the effects of an RS-based coating containing dried crude aqueous MPE at varying concentrations (0, 0.02, 0.1, 0.5, and 1.0% *w*/*w*) on the quality traits of frigate mackerel (*A. thazard*) slices during storage at 4 °C. 

## 2. Materials and Methods

### 2.1. Rice Flour and Starch Preparation

Here, a native underutilized short grain hard rice from Southern Thailand called Noui Khuea (NK) rice (*Oryza sativa* L.) was used. NK brown rice was obtained from a farm in Phra Phrom, Nakhon Si Thammarat, Thailand. To prepare rice flour, brown rice was ground in a dual-disk stone mill after being immersed in water at a proportion of 1:4 (*w*/*v*) for 6 h at 4 °C. The slurry was then put into a large cloth bag and pressed using a hydraulic press for 10 min. The moisture content of the wet-milled flour was subsequently reduced to around 13% by drying it at 60 °C in a hot-air oven. Using an MK 5087M Panasonic Food Processor (Shah Alam, Malaysia), the dry sample was pulverized into a fine powder and sieved through a 100-mesh sieve. Until it was required, rice flour was stored at −20 °C in ethylene–vinyl alcohol copolymer (EVOH) bags. The storage period was no longer than one month.

To prepare RS, rice flour was mixed in a 1:10 (*w*/*v*) ratio with 0.3% sodium hydroxide, stirred for 30 min, and left to stand at room temperature (27 to 29 °C) overnight. The precipitate was washed with distilled water after the cloudy supernatant was carefully drained. To ensure the supernatant was clear, the washing procedure was carried out 3 times. The precipitate was resuspended in distilled water and run through a steel strainer with a mesh size of 100. The resulting filtrate, referred to as rice starch (RS), was air-dried for 12 h at 40 °C [45].

### 2.2. Extraction of Mon-Pu (Glochidion wallichianum) Leaf Extract (MPE) 

Sample of dried Mon-pu leaf powder (2 g, moisture content was <10%; passed through a 25-mesh sieve) was extracted with 50 mL of distilled water. The mixture was continuously stirred for 15 h at 50 °C in a shaking water bath. Extract was filtered through a Whatman no.4 filter paper (Whatman International Limited, Maidstone, UK) [43] and referred to as crude MPE. According to the Folin–Ciocalteu colorimetric method [46], the crude MPE total extractable phenolic content was found to be roughly 11.34 g/L as gallic acid. Then, the crude MPE was subjected to freeze-drying (CoolSafe 55-4 Pro Freeze dryer, ScanLaf A/S, Allerød, Denmark). The freeze-dried sample was ground into powder using a stainless steel grinder and passed through a 60-mesh sieve before being incorporated into a coating solution. Different amounts of dried MPE were added to the coating solution to achieve final concentrations in MPE of 0, 0.02, 0.1, 0.5, and 1.0% *w*/*w*. The preservation power of a phenolic is often dose dependent. As a result, MPE content varied from low (0.02%) to high (1%). Because MPE concentration more than 1% can adversely affect the sensory of the sample from the preliminary test, the maximum concentration of MPE was set at 1%.

### 2.3. Preparation of Mackerel Slices and Coating Solution

The mackerel (*Auxis thazard*) with an average weight of 1.5–1.8 kg and a total length of 32 ± 2 cm were purchased from the local market in Thasala, Nakhon Si Thammarat, Thailand. Within 30 min, the mackerel were sent to the lab for food processing at Walailak University. The mackerel were promptly gutted, scaled, and washed twice in cold water (4 °C). A nylon screen was used to drain the prepared mackerel for 1 min in a cold room (4–5 °C) after it had been cut into slices measuring 1 × 5 × 2 cm (width × length × height). The slices were separated into 6 groups, which included: (1) Uncoated (soaked in distilled water), (2) coated with RS without MPE, (3) coated with RS + 0.02% MPE, (4) coated with RS + 0.1% MPE, (5) coated with RS + 0.5% MPE, and (6) coated with RS + 1% MPE. Using 50 g of RS and 1000 mL of distilled water, moderate stirring was conducted at room temperature before the mixture was heated to 80 °C for 30 min to create an aqueous solution of starch. Following gelatinization, 2 g/100 g (*w*/*w*, on a dry basis of the weight of starch) of glycerol was supplied as a plasticizer, and the resulting dispersion was exposed to additional mixing for 5 min. Then, MPE of different concentrations (0.02%, 0.1%, 0.5%, and 1% *w*/*w*, based on total extractable phenolic content) was added. After that, samples were homogenized for 5 min at 20,000 rpm. 

### 2.4. Coating Procedure and Storage of Coated Fish Slices

To create the coated fish slices, samples were gently dipped into the coating solutions for 2 min, and manually mixed every 30 s. In the same way as the treated samples, the uncoated control sample was submerged in distilled water. Samples were wrapped in shrink film and placed on a polystyrene foam tray. All the packaged samples were kept in a refrigerator at 4 ± 0.5 °C for 9 days and samples were taken at days 0, 3, 6, 7, 8, and 9 for analysis. The microbiological index was used to evaluate the storage period with a permissible limit of 6 log CFU/g. Each treatment had three sets of trays for the experimental replications (6 treatments × 3 repetitions = 18 samples). Replication was carried out three times for the analysis. The mackerel samples were immediately examined once the packages were opened.

### 2.5. Microbiological Analysis

Using the method outlined by Chaijan et al. [35], the total viable count (TVC) and total psychrophilic bacterial count (TPC) were measured and expressed as log10 colony forming units (CFU)/g.

### 2.6. Determination of Thiobarbituric Acid Reactive Substances (TBARS) and Gas Chromatography-Mass Spectrophotometry (GC-MS) Analysis for Propanal

The TBARS assay was carried out as per Buege and Aust’s instruction [47]. According to Chaijan et al. [35], GC-MS (7890B for the GC system and 7000D for the mass analyzer, Agilent, USA) was used to assess the formation of propanal in the fish samples.

### 2.7. Determination of Surface Color and Total Color Difference Value (ΔE)

A portable Hunterlab Miniscan/EX apparatus with 10 standard observers and illuminant D65 (Hunter Assoc. Laboratory; Reston, VA, USA) was used to measure the surface color of the samples, including L* (lightness), a* (redness/greenness), and b* (yellowness/blueness). A standard of white and black was used to calibrate the equipment. According to Vega-Gálvez et al. [48], the following equation was used to compute the ΔE:

(1) $$\Delta E=\sqrt{{\left(\Delta L*\right)}^{2}+{\left(\Delta a*\right)}^{2}+\;{\left(\Delta b*\right)}^{2}}$$

where ΔL*, Δa*, and Δb* are the differences between the color characteristics of the samples and those of the control at Day 0.

### 2.8. Determination of Heme Iron and Metmyoglobin

Heme iron and metmyoglobin contents were measured in accordance with Wongwichian et al. [10] and Chaijan [49], respectively. Using 20 mL of cold, 40 mM phosphate buffer (pH 6.8), minced fish (2 g) was homogenized for 10 s at 13,500 rpm. The mixture was then centrifuged at 3000× *g* (4 °C/ 30 min). Through a Whatman No. 1 filter paper, the supernatant was filtered. Employing the buffer as a blank, the absorbance of the isolated myoglobin solution was determined at 525 nm. Myoglobin, which has an iron content of 0.35%, was used to compute the heme iron content. By measuring the absorbance at 525 and 630 nm, the extracts were subsequently subjected to determine their metmyoglobin level. The ratio of A630 to A525 indicates the proportional amount of metmyoglobin.

### 2.9. Determination of pH, Total Volatile Base-Nitrogen (TVB-N), and Trimethylamine (TMA)

Minced fish (1 g) was homogenized with 10 mL of distilled water at room temperature at 13,400 rpm for 1 min. The pH was then determined using a pH meter (Cyberscan 500, Singapore). The Conway micro-diffusion assay was used to measure the contents of TVB-N and TMA [50]. 

### 2.10. Determination of Water Holding Capacity (WHC)

A fish sliced sample was weighed and placed between 2 pieces of Whatman No. 1 filter paper at the top and 3 pieces at the bottom. The sample was topped with a standard weight (5 kg), which was kept in place for 2 min. After that, the sample was taken out and weighed once again [35]. The following equation was used to determine WHC:

(2) $$\mathrm{WHC}\;(\%)=\left[\frac{\mathrm{Final}\;\mathrm{weight}}{\mathrm{Initial}\;\mathrm{weight}}\right]\;\times \;100\;$$

### 2.11. Determination of Texture

Using a TA-XT2i texture analyzer (Stable Micro Systems, Godalming, Surrey, UK) fitted with a spherical plunger (diameter 5 mm, depression speed of 60 mm/min), the hardness of a fish slice was measured using a double compression test [51]. Before measurement, the fish slice was tempered to room temperature.

### 2.12. Sensory Analysis

At Days 0 and 9, the likeness score of mackerel slices was assessed using a 9-point hedonic scale [35]. Scores ranged from 1 for “dislike extremely” to 9 for “like extremely”. The Institutional Ethics Committee approved the study, which was carried out in accordance with the Declaration of Helsinki’s criteria (WUEC-21-135-01). All participants in the study provided their informed permission.

Ten trained panelists, aged between 23 and 35, from the Department of Food Industry’s laboratory personnel (5 females and 5 males), assessed the sensory quality of the mackerel slices. The panelists regularly consumed the mackerel slices and Mon-pu and had no sensitivities to either food. The samples were acclimatized at room temperature before evaluation and were served on random three-digit number-coded plates. All panelists were asked to assess appearance, color, odor, and overall likeness.

### 2.13. Statistical Analysis

All experiments were carried out in triplicate, with the exception of the sensory analysis, which was performed by a panel of 10 participants. Data were subjected to analysis of variance (ANOVA). Comparison of means was carried out by Duncan’s multiple range test. Statistical analysis was performed using the Statistical Package for Social Science (SPSS 17.0 for windows, SPSS Inc., Chicago, IL, USA). It was declared significant when the probability value was *p* < 0.05. 

## 3. Results and Discussion

### 3.1. Changes in Microbiological Quality

**Figure 1:**
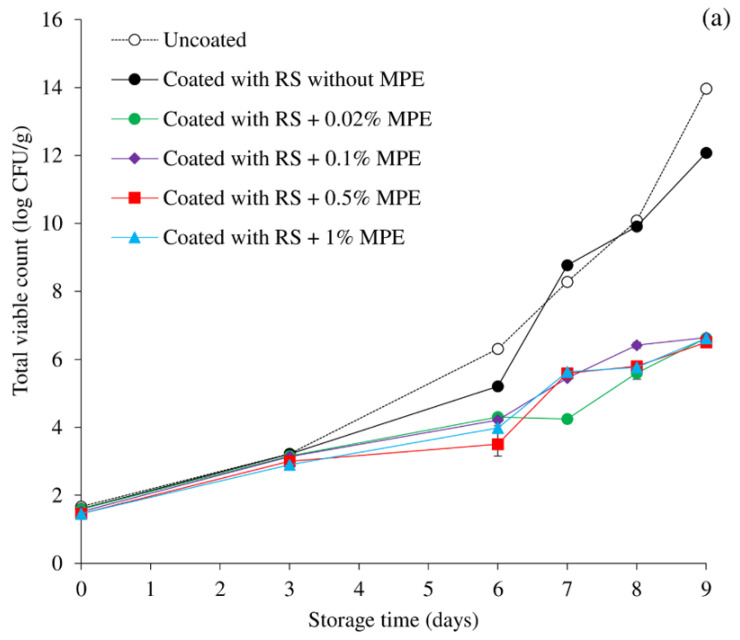

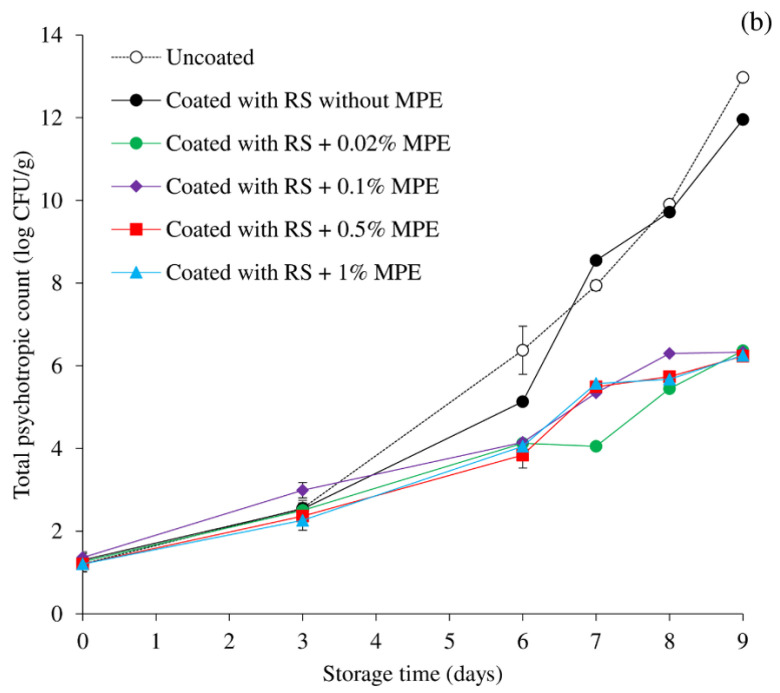
Changes in the total viable count (**a**) and total psychotropic count (**b**) of mackerel slices over the course of storage at 4 °C influenced by coating with rice starch (RS) containing Mon-pu extract (MPE) at different concentrations. Bars represent standard deviation from triplicate determinations.

Figure 1a,b shows the changes in the TVC and TPC of mackerel slices over the course of storage. There was no difference between the initial TVC and TPC of any treatment (*p* > 0.05); these values ranged from 1.46 to 1.66 log CFU/g and 1.20 to 1.36 log CFU/g, respectively, indicating the freshness of the fish. Typically, TVC < 4 log CFU/g sample suggests that the fish is suitable for consumption [52]. Sushi samples appear to be within the commonly accepted threshold limit of 6 log of CFU/g specified for ready-to-eat food products, despite the fact that the total bacterial contamination limits in sushi samples are not established [5,52]. Furthermore, Kulawik et al. [53] observed that, on the final day of their shelf-life, the total aerobic bacterial counts of ready-to-eat sushi dishes from six different manufacturers were typically between 5 and 6.5 log CFU/g. Therefore, the TVC of 6 log CFU/g was employed in this study as the microbiological limit. All samples showed increases in TVC and TPC with longer cold storage times, although coating with RS containing 0.02–1% MPE appeared to slow microbial growth in comparison to uncoated samples or coating without MPE. This may be related to the MPE’s antibacterial properties as well as the coating with RS, which may render the bacterial population dormant during sample preparation. Uncoated fish fillets had a shelf-life of ~6 days when taking into account the TVC and TPC of 6 log CFU/g, however coating without MPE can extend the shelf-life by about 7 days.

Based on the microbiological limit for fish consumption (6 log CFU/g), the shelf-life of the MPE-coated samples can be prolonged up to 9 days, regardless of the MPE concentration. Accordingly, coating mackerel slices with RS containing MPE at 0.02–1% effectively extended the shelf-life up to 9 days based on the microbial load. Regardless of concentration, MPE significantly influenced the slowing of microbial growth in the coated samples. Coatings, in general, aid in keeping various active ingredients included in their formulation, like antimicrobials, on the product surface for extended storage times, boosting their efficiency [25]. Gallic acid, methyl gallate, luteolin-6-C-d-glucopyranoside, luteolin-8-C-d-glucopyranoside, and apigenin-8-C-d-glucopyranoside are phenolic compounds with antibacterial effects that have been documented in MPE [38,40]. In crisp grass carp, silver carp, and sea bass fillets, polyphenol dip can prevent microbial development [35,37,54]. Gallic acid is frequently used due to its potent antioxidant activity as well as its antibacterial potential [55]. Gallic acid has also been found to be a natural phenolic cross-linker or plasticizer that can alter the mechanical properties of biological polymer materials, which promotes its usage as a food-packaging material [56].

### 3.2. Changes in Lipid Oxidation 

**Figure 2:**
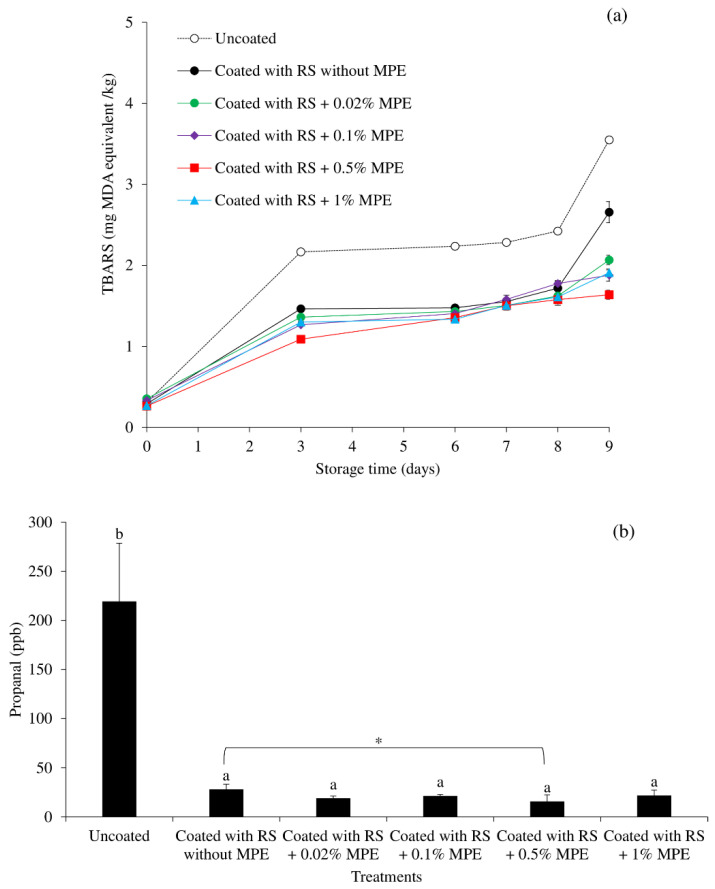
Changes in the thiobarbituric acid reactive substances (TBARS) of mackerel slices over the course of storage at 4 °C as influenced by coating with rice starch (RS) containing Mon-pu extract (MPE) at different concentrations (**a**), and the propanal content of mackerel slices coated with RS containing MPE at different concentrations at Day 9 of storage at 4 °C (**b**). Bars represent standard deviation from triplicate determinations. Different letters on the bars indicate significant differences (*p* < 0.05). The difference between coated samples with a *p* < 0.05 is indicated by an asterisk (*).

Lipid oxidation of the mackerel fillets given by TBARS during storage is illustrated in Figure 2a. All sample TBARS levels had a tendency to rise throughout the course of storage. This was consistent with the findings of Maqsood and Benjakul [57] and Chaijan et al. [35], who reported on the elevated TBARS contents of uncoated and WPI-phenolic extract coated Asian sea bass slices during chilled storage, respectively. In order to improve the retention of seafood quality by preventing both microbial activity and lipid oxidation events, Trigo et al. [17] combined gelatin packaging films with various natural preservative compounds from a variety of sources, such as oregano and rosemary extracts, oregano oil, tea polyphenols, or a microalga protein concentrate. According to the findings, at all storage times, the coated samples—especially those with MPE—showed lower TBARS values than the uncoated samples. TBARS levels in uncoated samples increased throughout time, peaking at 3.5 mg MDA equivalent/kg at the end of storage. By reducing oxygen diffusion, coating fish can slow the oxidative rate while the formulated phenolic antioxidants also act synergistically to slow lipid oxidation [35]. Gallic acid, methyl gallate, orientin, isoorientin, vitexin, and other antioxidant polyphenols are abundant in MPE [40], which may act as a free radical scavenger and chelator to delay the oxidation of lipids in mackerel fillets, which are rich in pro-oxidant iron in dark muscle, during storage. Reactive oxygen species suppression and chelation action are both necessary for polyphenol antioxidant activity [58]. Gallic acid, a plant phenolic antioxidant, has been used in combination with chitosan as a coating material to inhibit the rise of TBARS in refrigerated tilapia fillets [34,59]. At the end of storage (Day 9), coating without MPE displayed the highest TBARS, whereas coating with 0.5% MPE displayed the lowest value. The TBARS increased (*p* < 0.05) when MPE was present in concentrations lower or greater than 0.5%. Thus, the recommended amount of MPE to add to the RS coating is 0.5%. According to Wongnen et al. [38], MPE may behave as a pro-oxidant at high concentration since phenolics are redox-active compounds with both pro-oxidant and antioxidant effects on biological systems [58]. The effective concentration of MPE in a given product must therefore be optimized.

To confirm the deceleration of lipid oxidation of coating with RS containing MPE, the aldehydic lipid oxidation product propanal was compared at Day 9 of storage (Figure 2b). Aldehydes are frequently the main factors causing rancidity development in foods rich in unsaturated fatty acids, notably seafood, and they are known to be detrimental to human health [35]. The findings showed that coating alone can, to some extent, inhibit propanal accumulation, but coating in the presence of MPE can, in a concentration-dependent manner, effectively delay propanal production. The lowest amount of propanal was found in a sample coated with 0.5% MPE, and it rose at concentrations greater or lower than this one, similar to the TBARS contents (Figure 2a).

### 3.3. Changes in Pigment and Color

**Figure 3:**
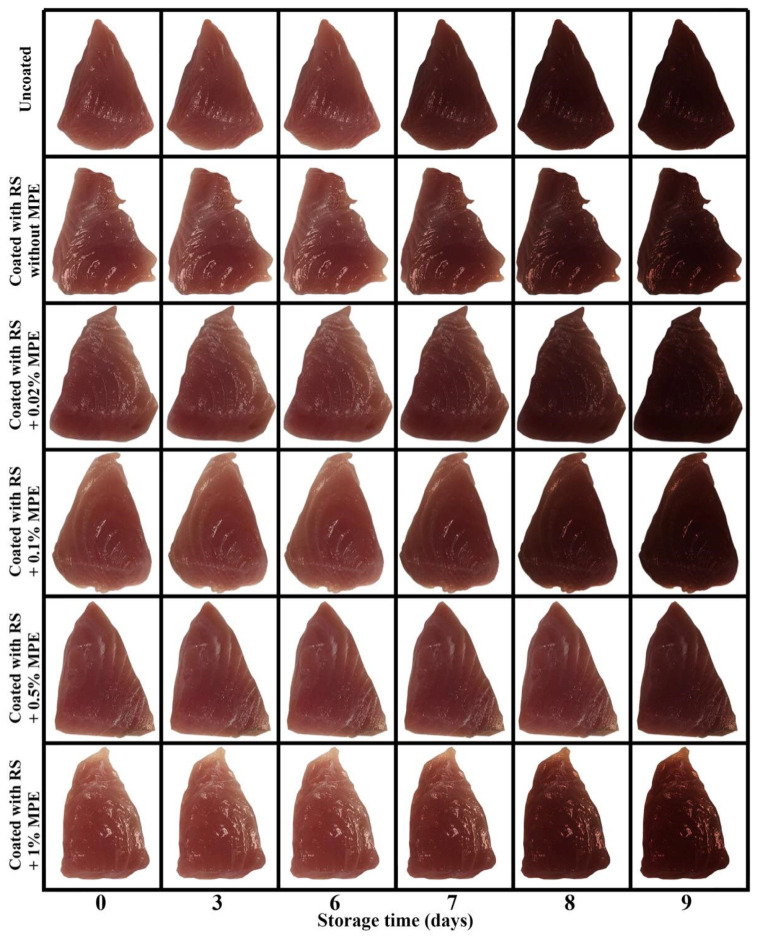
Changes in the appearance and color of mackerel slices over the course of storage at 4 °C as influenced by coating with rice starch (RS) containing Mon-pu extract (MPE) at different concentrations.

Figure 3 illustrates the appearance and color of mackerel slices as influenced by coating with and without MPE at different concentrations. From the observation, the initial color of all mackerel slices was red in color and seemed to remain unchanged over 6 days of storage. However, the effect of coating and the incorporation of MPE on the discoloration can be observed from Day 7 until the end. Uncoated slices turned brown at Day 7 and the intensity increased up to 9 days. Coating without MPE seemed to retard the discoloration to some extent but the discoloration can also be observed at Day 7–9, likewise the coating with 0.02–1% MPE. The incorporation of 0.5% MPE was the most effective treatment in retarding the discoloration of mackerel slices as the darkening was minimized. However, at higher concentration, MPE promoted the darkening of the slice. 

**Figure 4:**
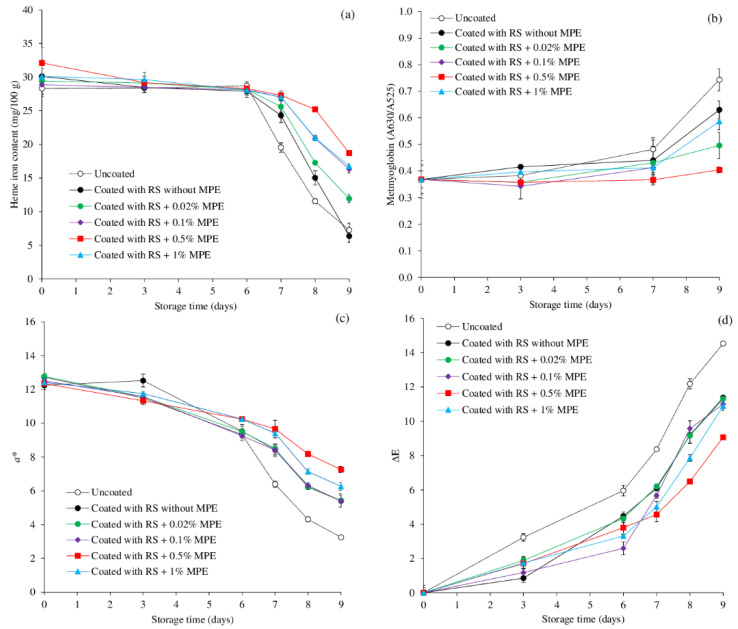
Changes in heme iron content (**a**), metmyoglobin (**b**), a* value (**c**), and total color difference ΔE (**d**) of mackerel slices over the course of storage at 4 °C as influenced by coating with rice starch (RS) containing Mon-pu extract (MPE) at different concentrations. Bars represent standard deviation from triplicate determinations.

To understand the mechanism in discoloration of mackerel slices as influenced by coating with and without MPE at different concentrations, the changes in heme iron content, metmyoglobin content, a* value, and ΔE were monitored as shown in Figure 4a–d. 

Changes in the heme iron contents of the mackerel slices during chilled storage are depicted in Figure 4a. All sample heme iron contents progressively fell during the course of the first 6 days of storage before rapidly declining after that. At the final stage, the lowest heme iron content, implying the highest degree of heme protein degradation, was observed in the uncoated sample and the coated sample without MPE, accounting for 74–79% reduction (*p* < 0.05). The fish coated with the RS containing MPE, notably at 0.5% (42% reduction), had the maximum amount of heme iron persisting, suggesting having the most preventive effects against heme protein decomposition. In general, the accumulation of non-heme iron and the oxidation of lipids and proteins in fish muscle are related to the decrease of heme iron concentration [10,14]. Maqsood, and Benjakul [57] reported that the heme iron concentration of the bled sea bass slice did not change during the first 6 days of storage; however, it gradually decreased until the completion of the storage time. The release of non-heme iron from a heme breakdown caused the heme iron concentration to drop as storage time increased. Alternately, the iron released can promote lipid oxidation. The primary mechanism by which non-heme iron has been linked to its ability to induce lipid oxidation is through the breakdown of lipid hydroperoxides to produce free radicals that can take hydrogen atoms from fatty acids and begin the process [14]. A decrease in heme iron content seemed to correlate with the lipid oxidation (Figure 2a) and the discoloration as indicated by increased metmyoglobin content (Figure 4b), decreased surface a* value (Figure 4c) and increased ΔE (Figure 4d). Thus, the degradation and oxidation of myoglobin resulted in the discoloration of mackerel slices during storage. 

After 9 days of storage, the uncoated fish slice had a higher metmyoglobin content than the coated samples (Figure 4b), indicating that the uncoated fish slice had a higher rate of myoglobin oxidation. These were connected to lipid oxidation (Figure 2a). According to Faustman et al. [60], lipid and myoglobin oxidations frequently co-occur. Aldehydic lipid oxidation products, according to Suman and Joseph [61], potentially promote myoglobin oxidation. Typically, during the preparation and storage of fish, lipid deterioration, protein oxidation, and discoloration are connected [10,14]. A similar pattern in the delay of myoglobin oxidation was present in the coated fish slices that had either RS alone or RS combined with MPE but the presence of MPE seemed to be more effective. At the end of storage, the sample coated with RS containing 0.5% MPE was the most effective in suppression the metmyoglobin formation, followed by those with 0.02–0.1% MPE, and 1% MPE, respectively. The antioxidant power of phenolic compounds in MPE combined with the oxygen barrier by RS coating led to the lower degree of metmyoglobin formation.

As a result of metmyoglobin formation, heme protein degradation, and other discoloration pathways, the surface a* of mackerel slices changed considerably. Although the color from the observation in Figure 3 seemed to be stable during the first 6 days of storage in all treatments the instrumental a* values decreased throughout the course of storage (Figure 4c). Coating with RS in the presence of MPE, particularly at 0.5%, can effectively retard the loss of redness. Uncoated sample had the lowest a* value at the end of storage (Figure 4c). When the total color difference (ΔE) was calculated in order to compare the total color of the initial slice with treatment at all-time points. The ΔE of the uncoated mackerel slice tended to increase more quickly than the coated fish samples during storage (*p* < 0.05), indicating the effectiveness of the RS-based coating with and without phenolic extracts in delaying the total color changes of the mackerel slice during storage. So, uncoated sample had the most pronounced discoloration while the least discoloration was found in coated sample in the presence of 0.5% MPE (Figure 4d). This was most likely due to the antioxidant capabilities of MPE, which were confirmed by its strong reducing power and free radical scavenging activity [43]. These properties were able to prevent the conversion of oxymyoglobin to metmyoglobin by offering electrons and reducing ability. This was in line with the findings of Chaijan et al. [35], who discovered that a whey protein isolate coating containing polyphenol extract from ginger, lemongrass, or green tea during cold storage caused a slower increase in the ΔE values of Asia sea bass steak. Usually, protein denaturation, protein/lipid breakdown, and pigment oxidation of the fish slice surface contribute to the color shift by absorbing and dispersing light. The primary factor affecting meat color during storage is the oxidation of red myoglobin to brown metmyoglobin, which results in a reduced tone of redness in the flesh [35]. Furthermore, melanoidins produced by the amine–carbonyl process, which are formed as a result of lipid oxidation, might cause tuna to turn dark [2]. Free radical damage to the conjugated double bonds of pigments caused a loss of color as well [2]. As shown by the lower a* value of the uncoated samples, lipid oxidation may therefore be another potential cause of the darkening of the slices. Overall, coating mackerel slices with RS containing 0.5% MPE demonstrated the potential to preserve the quality of the fish by minimizing the production of secondary lipid oxidation products, which may further cause oxymyoglobin to oxidize through a chain reaction of free radicals [2]. Altogether, the coating with RS containing MPE can reduce the amount of lipid oxidation, heme protein breakdown, metmyoglobin production, and discoloration.

### 3.4. Changes in pH and Off-Flavor Development 

**Figure 5:**
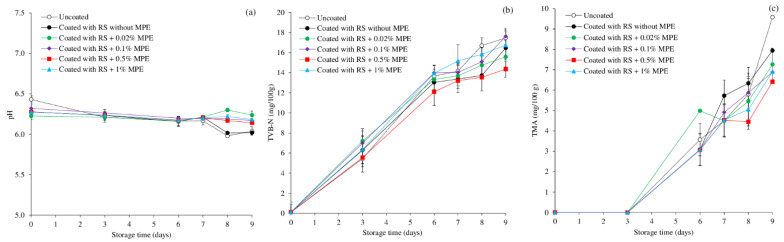
Changes in pH (**a**), total volatile base-nitrogen (TVB-N) (**b**), and trimethylamine content (TMA) (**c**) of mackerel slices over the course of storage at 4 °C as influenced by coating with rice starch (RS) containing Mon-pu extract (MPE) at different concentrations. Bars represent standard deviation from triplicate determinations.

The pH of the fish can change as a result of biochemical and chemical processes and microbiological development during storage. Figure 5a depicts how the pH of the mackerel slices changed throughout storage. The initial pH of uncoated fish was 6.4 and it was reduced to around 6.2 when MPE was incorporated at all concentrations (Figure 5a). The pH of the uncoated sample and fish coated with RS without MPE gradually decreased during storage, whereas the mackerel slice coated with RS containing MPE at 0.1–1% had negligible changes. This indicated the coating’s effectiveness in delaying the development of basic and acidic molecules as well as maintaining the mackerel’s potential to act as a buffer during storage. However, at low concentration, MPE at 0.02% tended to render the slice with increased pH from Day 6 to Day 9. 

Numerous volatile substances, including ketones, alcohols, dimethyl sulfide, dihydrogen sulfide, aldehydes, TMA, and organic acids, are produced when fish spoils [62]. Unwanted flavors are created by endogenous and microbial proteases, which result in total volatile compounds. One of the most commonly used markers to assess the level of fish spoilage brought on by bacteria and autolytic enzymes is the TVB-N assay, which comprises TMA, ammonia, dimethylamine, and other volatile basic nitrogenous substances [37,63,64]. The TVB-N contents in all samples were detected at very low concentration at the beginning, suggesting the freshness of fish (Figure 5b). This was in agreement with the microbial quality (Figure 1). With increasing storage time, the TVB-N of all samples increased to a varying degree depending on the treatment, but the acceptable limit of 25 mg/100 g for acceptable product quality was not exceeded [65]. RS-based coating with MPE, especially at 0.5% showed the most inhibition against TVB-N and it correlated well with the lowest microbial growth (Figure 1). The higher microbial inactivation of the RS coating and MPE was the reason for the coated samples’ lower TVB-N levels (Figure 1a,b). By reducing the amount of oxygen available to aerobic bacteria inside the fish slice, coating fish with RS alone may indirectly decrease microbial growth but the incorporation of MPE with phenolic antimicrobial effect can improve the antimicrobial effect as well as to suppress the endogenous enzyme activities. The TVB-N levels of silver carp and sea bass slices have both been reported to be inhibited by polyphenols [35,37].

Changes in TMA as a key indicator of fishy odor development are shown in Figure 5c. TMA was not detected in all samples during the first 3 days. The formation of TMA progressively increased thereafter, till the end of storage. At Day 9, uncoated fish showed the highest TMA, followed by coated fish without MPE. With MPE, the TMA values were lowered than the uncoated and coated without MPE. Among the coated group, the lowest TMA value was found in fish coated with RS containing 0.5% MPE as observed for TVB-N (Figure 5b). Results indicated that coating alone could delay the production of TMA to some degree. However, the incorporation of MPE at the optimal concentration, i.e., 0.5% can reduce the TMA accumulation more effectively. Although no official TMA limit has been established, acceptable TMA levels for specific fish species, such as 5–10 mg/100 g for sardines, have been suggested [66]. The TMA levels found in this instance were less than 10 mg/100 g, indicating that all samples were acceptable over the entire storage period.

### 3.5. WHC, Texture, and Sensory Aspects 

**Figure 6:**
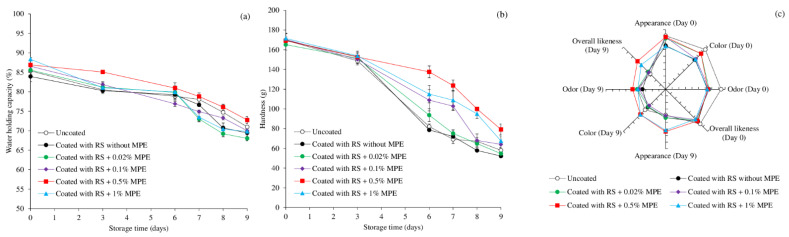
Changes in water holding capacity (**a**), hardness (**b**), and likeness scores (**c**) of mackerel slices over the course of storage at 4 °C influenced by coating with rice starch (RS) containing Mon-pu extract (MPE) at different concentrations. Bars represent standard deviation from triplicate determinations. Using a 9-point hedonic scale, 10 trained panelists evaluated the sensory quality on Days 0 and 9 of storage.

All samples experienced water loss during cold storage, as evidenced by the lower WHC (Figure 6a). This was most likely caused by the structural changes in muscle proteins brought on by cold-induced denaturation, protein disintegration, protein oxidation, and interactions between proteins and oxidized lipid, aldehyde, amine, and oxide compounds, which led to the loss of WHC [35]. The destabilization of carbohydrate–water interaction between coating material and fish muscle could be another possible cause of drip loss. However, WHC in all samples was more than 70% at the end of the storage period (Figure 6a). Overall, the WHC of the mackerel slices appeared to be stabilized more efficiently after the coating with 0.5% MPE. According to Galus and Kadzinska [67], this coating may increase the quality and extend the shelf life of foods by preventing the passage of moisture, oxygen, and other solutes. A barrier that prevents the flow of both moisture and oxygen can be advantageous for seafood [68,69]. As a result, the water binding ability, antioxidation, and antibacterial activities of each coating component may be the reason for the protective effectiveness of the RS-based coating with MPE for fish slice WHC.

Figure 6b displays changes in the fish slice textural characteristics as measured by hardness. At Day 6, all samples’ hardness dramatically decreased, and a little decline in hardness persisted through the conclusion of storage (Figure 6b). The coated samples, especially those with MPE at 0.1–1%, tended to retain more hardness than the uncoated sample and coated samples with and without 0.02% MPE, demonstrating the edible coating’s ability to preserve textural integrity when combined with optimum MPE. The fish coated with RS containing 0.5% MPE appeared to have better textural properties over the course of storage. The cross-linking of food constituents, particularly protein–protein, protein–phenolics, and protein–water, may contribute to the preservation of the mackerel slices’ textural qualities. The coating with RS and crude phenolic extracts may operate to inhibit microbial development and natural proteolytic enzymes, delaying the weakening of the muscle. Both in vitro and in fish products, polyphenols have been shown to have protease inhibitory properties. Trypsin activity has been reported to be inhibited by plant polyphenols (such as caffeic acid, cinnamic acid, gallic acid, chlorogenic acid, feruric acid, quinic acid, eugenol, and catechol) and their activities were influenced by the composition and concentration of the phenolic [70]. 

Figure 6c shows the likeness scores for the mackerel slices assessed at Days 0 and 9 using a 9-point hedonic scale. These likeness scores include appearance, color, odor, and overall likeness. Generally, the acceptability of mackerel slices or sashimi is governed by the color of slices. At Day 0, the uncoated sample tended to have the highest liking score for all attributes whereas the lowest score was obtained for RS coated fish with 1% MPE coating. Coating with RS containing 0.5% MPE had no different appearance, color, and overall likeness scores at Day 0 but it reduced the odor score (*p* < 0.05). The odor scores of all coated samples were not different, suggesting that the concentrations of MPE at 0.02–1% provided the same odor intensity in mackerel slices. The decreased liking score may be due to MPE interference with the usual odor of mackerel slices, which alters their distinctive odor.

All liking values for every sample after 9 days of storage were lower than they were on Day 0 (*p* < 0.05). For appearance and color, samples coated with RS containing 0.5% and 1.0% MPE had the highest liking score (*p* < 0.05). For odor and overall likeness, samples coated with RS containing 0.5–1% MPE tended to obtain higher scores than uncoated samples (*p* < 0.05). Overall, 0.5% MPE incorporated coating produced slices with higher sensory aspects at the end of storage and the score of liking exceeded 5, which is regarded as satisfactory for consumption [35]. This was due to the ability of coating materials to stabilize color, lipid oxidation, flavor, and texture as well as the microbial growth of the slices.

## 4. Conclusions

Mackerel slices can be preserved during chilled storage by using MPE-enriched RS edible coating. An effective biopreservative coating made of RS and MPE, especially at 0.5%, can be used to prevent microbial growth, lipid oxidation, myoglobin redox instability, heme protein degradation, discoloration, drip loss, texture softening, the development of an unpleasant odor, and sensory defects in refrigerated mackerel slices. The mackerel slice coated with RS combined with 0.5% MPE was able to preserve the microbiological quality below the allowable limit of 6 log CFU/g for 9 days, as opposed to the uncoated sample, which had a shelf-life of 6 days. However, from a consumer’s perspective, there are concerns about the safety of edible coatings, and from an industrial standpoint, there are issues about the high cost of edible polymers that need to be addressed. It is conceivable that advancements in edible coatings, using inexpensive food processing by-products as raw materials for formulation of edible coatings, combined with other preservation techniques, with the help of bioactive compounds, can be used to increase the shelf-life with safety for fish slices and other muscle foods.

## Data Availability

Data sharing is not applicable.
